# Three-Dimensional Analysis of Eyelid Morphological Outcomes After Hughes Flap Reconstruction for Full-Thickness Lower Eyelid Defects

**DOI:** 10.1007/s00266-026-05757-8

**Published:** 2026-05-15

**Authors:** Sitong Ju, Viola T. Rack, Julia K. Nosch, Senmao Li, Ludwig M. Heindl, Wanlin Fan, Alexander C. Rokohl

**Affiliations:** 1 Department of Ophthalmology, University of Cologne, Faculty of Medicine and University Hospital Cologne, Kerpenerstr. 62, 50937 Cologne, Germany; 2 Department of Ophthalmology, PLA General Hospital, Beijing, 100039 China; 3 Center for Integrated Oncology (CIO) Aachen –Cologne – Bonn – Duesseldorf, Cologne, Germany; 4 Department of Ophthalmology, Tongji Hospital, Tongji Medical College, Huazhong University of Science and Technology, Wuhan, China

**Keywords:** Hughes procedure, Lower eyelid tension, Distraction test, Weighted eyelid hook, Three-dimensional stereophotogrammetry, Anatomical landmarks

## Abstract

**Background:**

The standard technique used for full-thickness reconstruction of the lower eyelid is the Hughes tarsoconjunctival flap. Despite its widespread use, objective postoperative assessments of eyelid morphology and tension remain rare.

**Methods:**

This cross-sectional clinical study included patients who underwent the Hughes procedure at the Department of Ophthalmology, University of Cologne. Eyelid morphology was evaluated using the VECTRA M3 stereophotogrammetry system in neutral position, manual downward traction, and standardized hook distraction with a 15.9 g eyelid hook. Seventeen anatomical landmarks were identified per eye, measuring eight linear distances, two angles, and eyelid area. Operated and non-operated eyes were compared.

**Results:**

Sixteen patients were included; 75% had basal cell carcinoma. In neutral position, operated eyes showed significantly larger lateral lid distances (*p* = 0.004), upper lateral lid distances (*p* = 0.008), and lateral canthal angles (*p* = 0.010). Under manual distraction, the lateral canthal angle remained larger (*p* = 0.003), while lateral lid mobility was reduced (*p* = 0.039). Hook distraction revealed smaller length and area changes in operated eyes (*p* ≤ 0.023, respectively), indicating increased tension. Subgroup analysis showed these differences were more pronounced in patients with defects >50% of eyelid length. In small defects, differences persisted but were less marked.

**Conclusions:**

The Hughes procedure increases lower eyelid tension and reduces tissue compliance, particularly in the temporal region and in large defects. Standardized tension testing, such as weighted hook distraction, may improve postoperative assessment and guide surgical refinements.

**Level of Evidence III:**

This journal requires that authors assign a level of evidence to each article. For a full description of these Evidence-Based Medicine ratings, please refer to the Table of Contents or the online Instructions to Authors www.springer.com/00266.

## Introduction

Basal cell carcinoma (BCC), squamous cell carcinoma (SCC), and melanoma are the most common malignant tumors affecting the eyelid, with BCC being the most prevalent [1, 2]. Surgical excision remains the gold standard for treatment but frequently results in substantial eyelid defects necessitating reconstructive intervention [3].

Following resection of these lower eyelid tumors, primary direct closure is generally the preferred method for reconstructing full-thickness defects, provided sufficient tissue is available to allow for tension-free approximation [4]. However, for larger defects or cases unsuitable for direct closure, alternative reconstructive techniques are required. These include tarsoconjunctival flaps, free tarsoconjunctival grafts, and single-stage advancement procedures, among others [2, 5, 6].

Among these approaches, the Hughes tarsoconjunctival flap, commonly known as the Hughes procedure, is widely regarded as a reliable and versatile option for lower eyelid reconstruction [7]. This two-stage technique involves advancing a tarsoconjunctival flap from the upper eyelid to reconstruct the posterior lamella of the lower eyelid, followed by anterior lamellar reconstruction using skin grafts or local flaps [8]. The Hughes procedure offers several advantages, including restoration of both functional support and satisfactory aesthetic outcomes, particularly in defects involving significant loss of both anterior and posterior lamellae [5, 9, 10]. Furthermore, it can be combined with other reconstructive methods to extend its applicability to larger or more complex defects [1].

Despite its well-established efficacy, the Hughes procedure is not without limitations. A major concern lies in the postoperative morphological changes of the reconstructed lower eyelid, which can potentially impact eyelid contour, mobility, and overall function. These alterations are especially pronounced in cases involving extensive defects, where tissue tension and anatomical distortion may lead to suboptimal functional and cosmetic results [11]. Such changes underscore the need for comprehensive evaluation and long-term follow-up of patients undergoing this procedure.

While the Hughes flap has been extensively employed in clinical practice with high success rates [1, 7, 12], there is a notable paucity of studies investigating its long-term morphological and functional outcomes. In particular, quantitative assessments of postoperative eyelid changes remain scarce, limiting the ability to objectively evaluate the full impact of this reconstruction technique.

To address this gap, the present study utilizes advanced three-dimensional (3D) imaging technology to systematically assess the postoperative morphological changes associated with the Hughes procedure. By providing objective and detailed measurements, this study aims to enhance the understanding of the long-term effects of the Hughes flap on eyelid morphology and function, ultimately informing surgical planning and optimizing patient outcomes in lower eyelid reconstruction.

## Methods

### Study Design and Subjects

This single-center, cross-sectional clinical study was approved by the Institutional Review Board of the Department of Ophthalmology, University of Cologne, and adhered to the principles of the Declaration of Helsinki (17-199). Written informed consent was obtained from all participants prior to inclusion.

Patients who had undergone a Hughes tarsoconjunctival flap procedure (2-staged standardized modified Hughes procedure) performed by Dr. Ludwig M. Heindl [1] at the Department of Ophthalmology, University of Cologne, between October 2013 and December 2020 were retrospectively identified. Patients were excluded if they had received bilateral Hughes procedures, underwent additional surgical interventions on the operated eye after the initial procedure, or had a postoperative follow-up period of less than three months. Further exclusion criteria included clinical signs of lower lid margin erythema, severe ectropion or entropion, eyelid retraction, trichiasis, lagophthalmos, pyogenic granuloma, or flap suture dehiscence. Patients with a history of uncontrolled systemic diseases or treatments known to affect ocular surface conditions, those receiving topical therapies unrelated to ocular surface inflammatory diseases, as well as individuals with a history of ocular trauma, prior ocular surgeries or laser treatments, contact lens wear, or pregnancy were also excluded. Additionally, patients who were unwilling or unable to cooperate during the examination were not considered for inclusion.

For all eligible patients, demographic and surgical data were collected, including sex, age at the time of surgery, clinical diagnosis, details of the surgical procedure, and the size and location of the excised tissue.

### Three-Dimensional Stereophotogrammetric Image Acquisition and Analysis

Three-dimensional images were acquired using the VECTRA M3 Imaging System (Canfield Scientific, Inc., New Jersey, USA) and analyzed with VAM software version 2.8.2. All image acquisitions were performed by an experienced operator following the manufacturer’s standardized protocols. Photographic examinations took place in a dedicated clinical photography room with consistent ambient lighting, avoiding natural light sources to ensure uniform imaging conditions. Patients were asked to remove any makeup and to secure their hair to prevent obstruction of the eyebrows. During imaging, patients maintained a neutral head position and a relaxed facial expression, with lips gently closed and eyes naturally open.

**Fig. 1:**
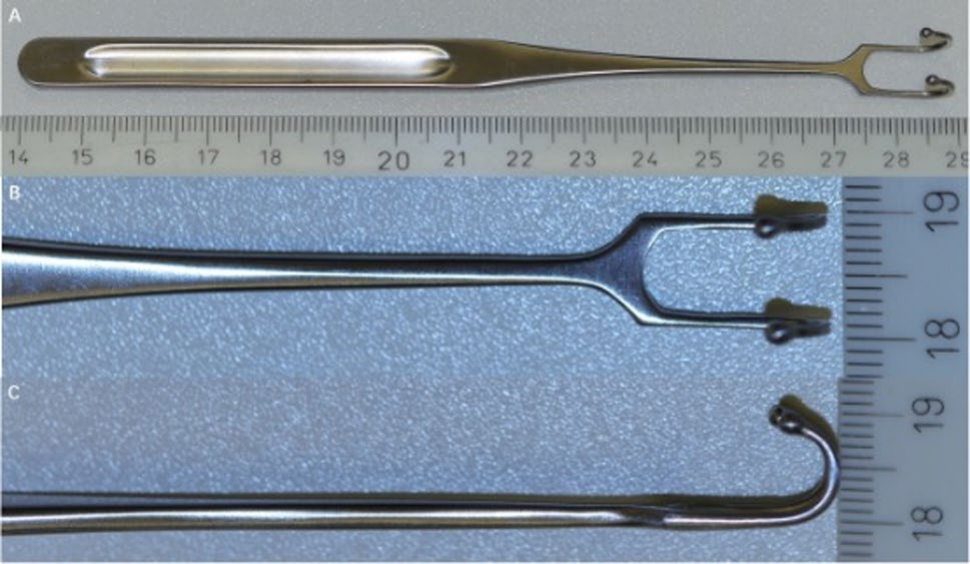
**A**–**C** Specifications of the 15.9 g stainless-steel eyelid hook used for standardized lower eyelid distraction. The instrument features a head width of 1.0 cm, body length of 15.0 cm, and thickness of 1.0 mm. The consistent application of the hook ensured reproducible mechanical distraction during three-dimensional imaging for postoperative morphological assessment

For each patient, five standardized three-dimensional images were obtained. Initially, an image was captured with the patient in a neutral position, gazing straight ahead with the eyes naturally open. Subsequently, the lower eyelid of the operated eye was gently pulled downward manually and photographed, followed by the same procedure on the contralateral, non-operated eye. Thereafter, a 15.9-g stainless-steel eyelid hook (1.0 cm head width, 15.0 cm body length, 1.0 mm thickness; Zhen Bang Medical Devices Co., Ltd., Anhui, China) was used to apply standardized downward traction on the lower eyelid of the operated eye, which was then photographed (Fig. 1) [13]. Finally, the same hook distraction was applied to the contralateral lower eyelid, and an image was acquired. All images were taken following the same procedural standards to ensure reproducibility and consistency across all subjects.

### Landmarks and Measurements

**Fig. 2:**
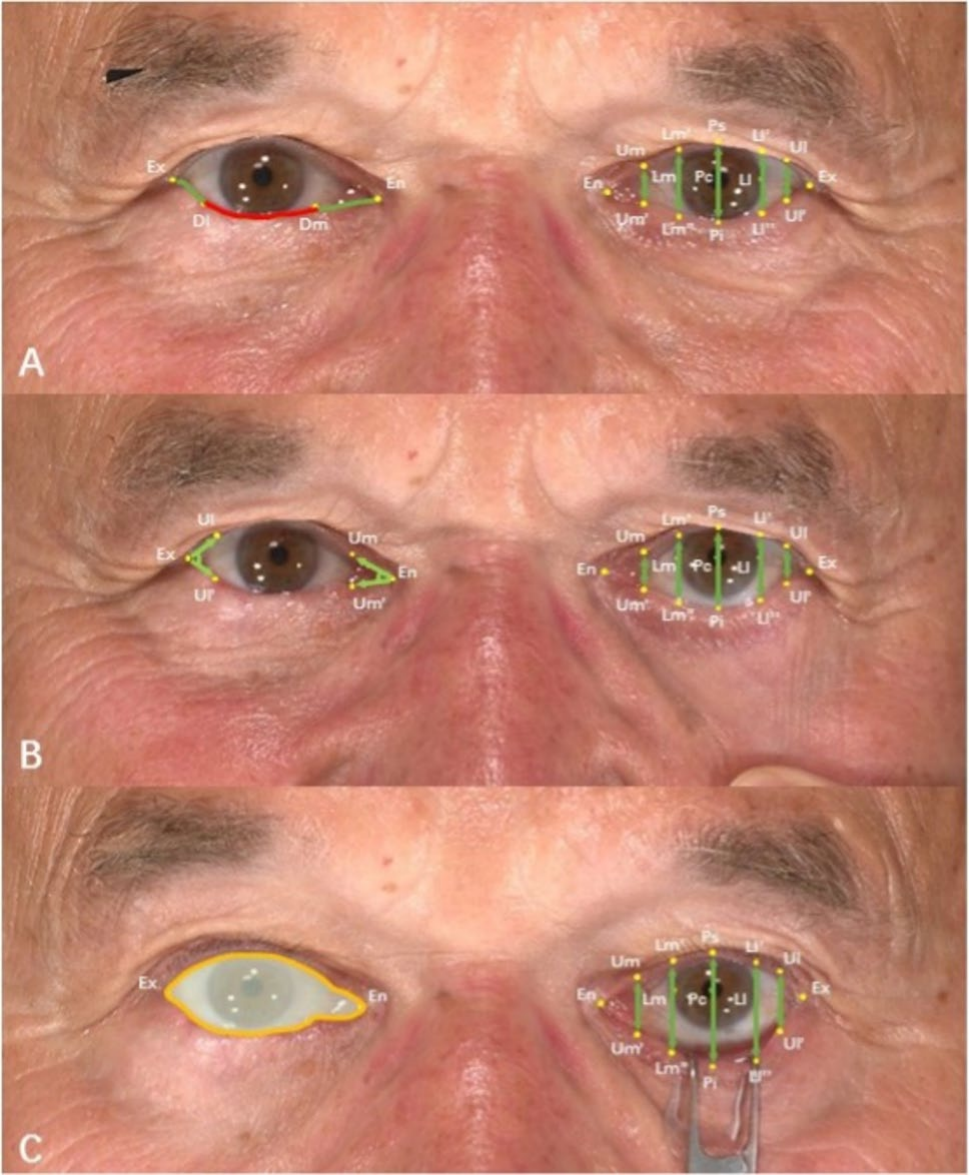
**A**–**C** Standardized three-dimensional analysis of the lower eyelid using seventeen ocular and periocular anatomical landmarks. Ten periocular anthropometric measurements were obtained under three conditions: **A** neutral position, **B** manual distraction, and **C** hook distraction. These measurements were used to quantitatively assess postoperative eyelid morphology following the Hughes tarsoconjunctival flap procedure

Based on established protocols from previous studies, seventeen ocular and periocular anatomical landmarks were digitally identified on each eye using the VAM software (Fig. 2, Table 1) [14, 15]. Initial reference points included the endocanthion (En), exocanthion (Ex), and the pupil center (Pc). Using the software’s ruler tool, additional landmarks were placed at the medial (Lm) and lateral (Ll) corneoscleral limbus of the pupil.

**Table 1:** Definitions and anatomical descriptions of the 17 periocular anthropometric landmarks used for three-dimensional eyelid analysis

Landmarks	Definition
En	Endocanthion, inner commissure of the palpebral fissure
Ex	Exocanthion, outer commissure of the palpebral fissure
Pc	Geometric center of the visible pupil
Lm	Medial corneoscleral limbus point horizontal to pupillary center
Ll	Lateral corneoscleral limbus point horizontal to pupillary center
Ps	Palpebrale superioris, vertically corresponding point to Pc at the upper palpebral margin on the lash line
Pi	Palpebrale inferioris, vertically corresponding point to Pc at the lower palpebral margin on the lash line
Lm’	Vertically corresponding point to Lm at the upper palpebral margin on the lash line
Lm”	Vertically corresponding point to Lm at the lower palpebral margin on the lash line
Um	Middle between En and Lm’ at the upper palpebral margin on the lash line
Um’	Middle between En and Lm” at the lower palpebral margin on the lash line
Ll’	Vertically corresponding point to Ll at the upper palpebral margin on the lash line
Ll”	Vertically corresponding point to Ll at the lower palpebral margin on the lash line
Ul	Middle between Ex and Ll’ at the upper palpebral margin on the lash line
Ul’	Middle between Ex and Ll” at the lower palpebral margin on the lash line
Dm	Medial margin of the eyelid defect
Dl	Lateral margin of the eyelid defect

From these primary landmarks (Lm, Pc, Ll), corresponding vertical points on the upper and lower eyelid margins were determined. These included Ll’, Ps, Lm’ (upper lid margin) and Ll’’, Pi, Lm’’ (lower lid margin). Subsequently, additional points were defined: the midpoint between Ex and Ll was marked as Ul and Ul’, while the midpoint between En and Lm was designated as Um and Um’.

The location of the eyelid defect was determined based on surgical records, specifically the documented size of the excised tissue. Accordingly, the medial margin of the defect was labeled Dm, and the lateral margin as Dl.

Using the Face Sculptor module of the VECTRA system, various periocular anthropometric measurements were calculated. These included the vertical distances between Um and Um’, Lm’ and Lm’’, Ps and Pi, as well as Ll’ and Ll’’. Further measurements encompassed the distance between Ul and Ul’, the length of the lower palpebral margin (LPMLm), and the medial and lateral canthal angles (MCA and LCA, respectively). The area enclosed between the upper and lower eyelids was also quantified.

Additionally, the horizontal length of the eyelid defect was measured by assessing the distance between Dm and Dl, refined using multiple reference points to enhance accuracy (Table 2). All measurements were obtained for both the operated and the contralateral non-operated eye for comparative analysis.

**Table 2:** List of the definitions of periocular anthropometric measurements used for quantitative analysis of lower eyelid morphology

Definitions	landmarks	Abbreviation
*Linear distances/length*		
Um–Um’ Distance	Um–Um’	UmD
Lm’–Lm’’Distance	Lm’–Lm’’	LmD
Palpebral fissure height	Ps-Pi	PFH
Ll’–Ll’’ Distance	Ll’–Ll’’	LlD
Ul–Ul’ Distance	Ul–Ul’	UlD
Lower palpebral margin length (more points)	Um’-Lm”-Pi-Ll”-Ul’-Ex including Dm and Dl as midpoints	LPMLm
Defect length (more points)	Including at least one more midpoint between Dm-Dl	DLm
*Angles*		
Medial canthal angle	Um-En-Um’	MCA
Lateral canthal angle	Ul-Ex-Ul’	LCA
*Area*		
Ocular surface area	Area between UPMLm and LPMLm	Area

### Statistical Analyses

All statistical analyses were performed using SPSS Statistics software, version 25.0 (IBM Corp., Armonk, NY, USA). The normality of data distribution was assessed using the Shapiro-Wilk test. Paired t-tests were used to compare periocular morphological parameters between the operated and contralateral non-operated eyes for normally distributed variables, while the Wilcoxon signed-rank test was applied for non-normally distributed data. A *p* value of less than 0.05 was considered statistically significant.

## Results

A total of sixteen patients (16 eyes) who underwent a Hughes tarsoconjunctival flap procedure at the Department of Ophthalmology, University of Cologne were included (Table 3). The mean patient age at the time of surgery was 70.40 years. The majority of patients were female (68.8%), and the mean postoperative follow-up duration was 25.08 months. Basal cell carcinoma represented the most common underlying pathology, accounting for 75.0% of cases.

**Table 3:** Demographic and clinical characteristics of patients undergoing Hughes flap reconstruction (n = 16)

Demographics	n = 16
Female, n (%)	11 (68.8 %)
Right eye, n (%)	8 (50.0 %)
Age, years (SD, range)	70.40 (9.07, 54-85)
Follow-up, months (SD, range)	25.08 (24.35, 4-81)
*Diagnosis*	
BCC, no. (%)	12 (75.0%)
SCC, no. (%)	2 (12.5%)
Melanoma, no. (%)	2 (12.5%)
*Defect fo lower eyelid*	
Size, mm (SD, range)	13.97 (6.17, 3.45-23.88)
Over 50%, no. (%)	7 (43.75%)

*SD* standard deviation, *BCC* basal cell carcinoma, *SCC* squamous cell carcinoma

### Comparison Between Operated and Non-operated Eyes Under Different Distraction Conditions

**Fig. 3:**
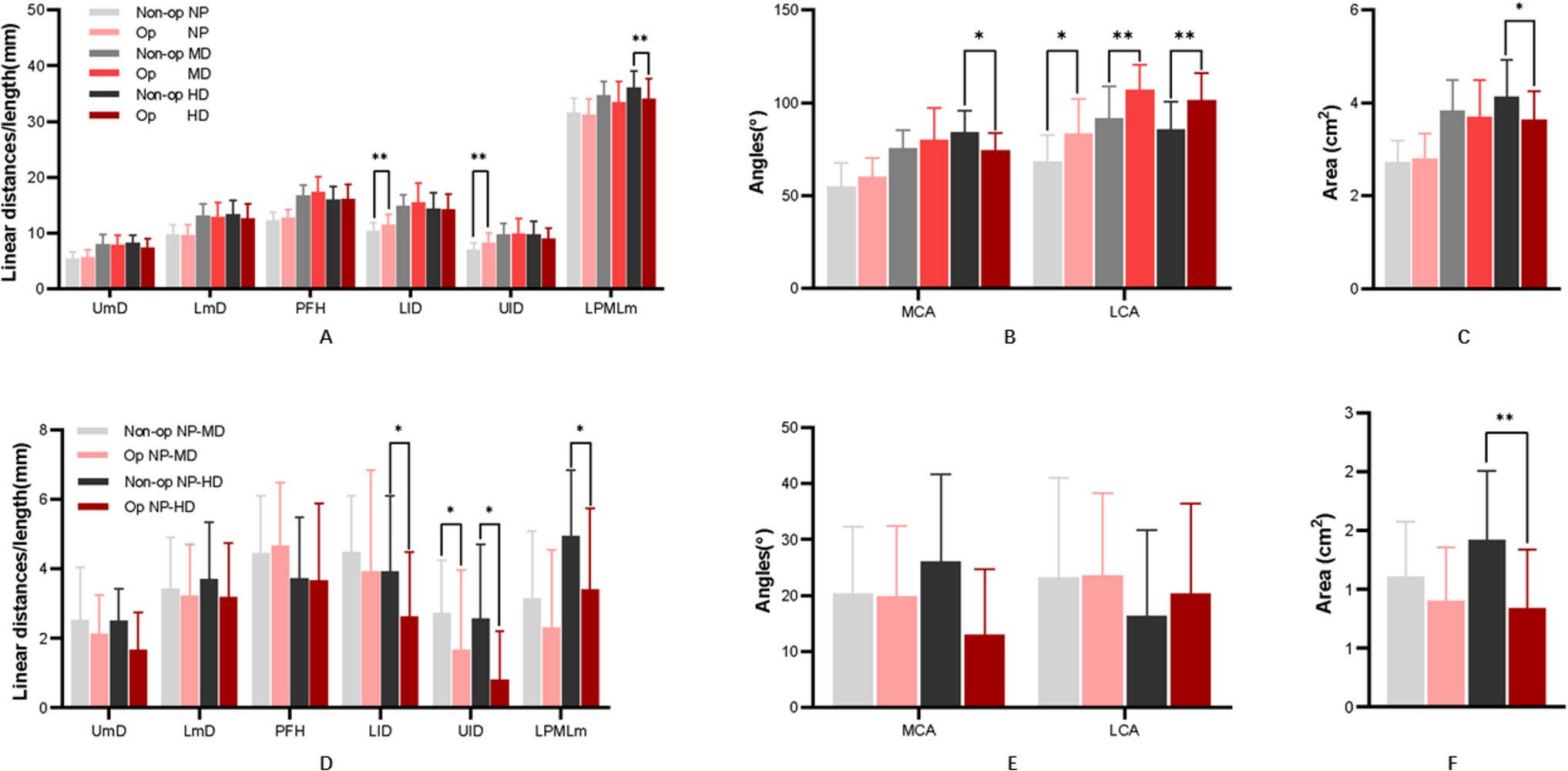
**A**–**F** Comparison of periocular measurements between the operated and contralateral non-operated eyes under different distraction conditions. **A**–**C** Absolute values of key parameters measured in neutral position (NP), manual distraction (MD), and hook distraction (HD). **D**–**F** Comparative analysis of the changes in measurements from neutral position to manual distraction (NP–MD) and hook distraction (NP–HD), highlighting differences in eyelid mobility and contour adaptation. *NP* neutral position, *MD* manual distraction, *HD* hook distraction. *p < 0.05; **p < 0.01; ***p < 0.001

Under the neutral position (NP), several periocular measurements were significantly larger on the operated side compared to the contralateral non-operated eye. Specifically, the lateral lid distance (LlD) measured 11.52 ± 1.85 mm on the operated side versus 10.37 ± 1.46 mm on the non-operated side (*p* = 0.004). Similarly, the upper lateral lid distance (UlD) was greater in the operated eye (8.18 ± 1.77 mm) compared to the non-operated eye (7.03 ± 1.21 mm, *p* = 0.008). The lateral canthal angle (LCA) was also significantly increased on the operated side (83.54 ± 18.49° vs. 68.40 ± 14.15°, *p* = 0.010). In contrast, measurements on the nasal side, including the upper medial distance (UmD), lower medial distance (LmD), palpebral fissure height (PFH), medial canthal angle (MCA), lower eyelid length (LPMLm), and the interpalpebral area, showed no significant differences between operated and non-operated eyes (Fig. 3A–C).

Under manual distraction (MD), the lateral canthal angle (LCA) remained significantly larger in the operated eye (107.14 ± 13.60°) compared to the non-operated side (91.62 ± 17.16°, *p* = 0.003). No other parameters showed significant differences under this condition (Fig. 3A–C). However, when assessing the degree of change from neutral to manual distraction (NP–MD), the operated eyelid exhibited significantly less displacement compared to the non-operated side, most notably in the upper lateral lid distance (UlD), which increased by only 1.68 ± 2.29 mm in the operated eye versus 2.73 ± 1.52 mm in the non-operated eye (*p* = 0.039) (Fig. 3D–F).

Under hook distraction (HD), most parameters were smaller on the operated side compared to the contralateral eye. Nevertheless, the LCA remained significantly larger in the operated eye (101.56 ± 14.54° vs. 85.62 ± 14.90°, *p* = 0.004) (Fig. 3A–C). Similar to the NP–MD comparison, the operated eyelid showed reduced mobility from neutral to hook distraction (NP–HD). Specifically, lateral side measurements such as LlD (2.63 ± 1.86 mm vs. 3.92 ± 2.19 mm, *p* = 0.023) and UlD (0.81 ± 1.41 mm vs. 2.56 ± 2.14 mm, *p* = 0.015) demonstrated significantly smaller increases in the operated eye. Furthermore, the increase in lower eyelid length (LPMLm) was significantly less on the operated side (3.41 ± 2.35 mm vs. 4.95 ± 1.90 mm, *p* = 0.023), as was the enlargement of the interpalpebral area (0.84 ± 0.50 cm^2^ vs. 1.42 ± 0.59 cm^2^, *p* = 0.002) (Fig. 3D–F).

### Comparison of Operated and Non-operated Eyes in Relation to Eyelid Defect Size Under Different Distraction Conditions

Among the included patients, seven cases (43.75%) presented with eyelid defects involving more than 50% of the lower eyelid length. In this subgroup, most periocular measurements were larger on the operated side compared to the contralateral non-operated eye, particularly in the neutral position (NP) and under manual distraction (MD).

**Fig. 4:**
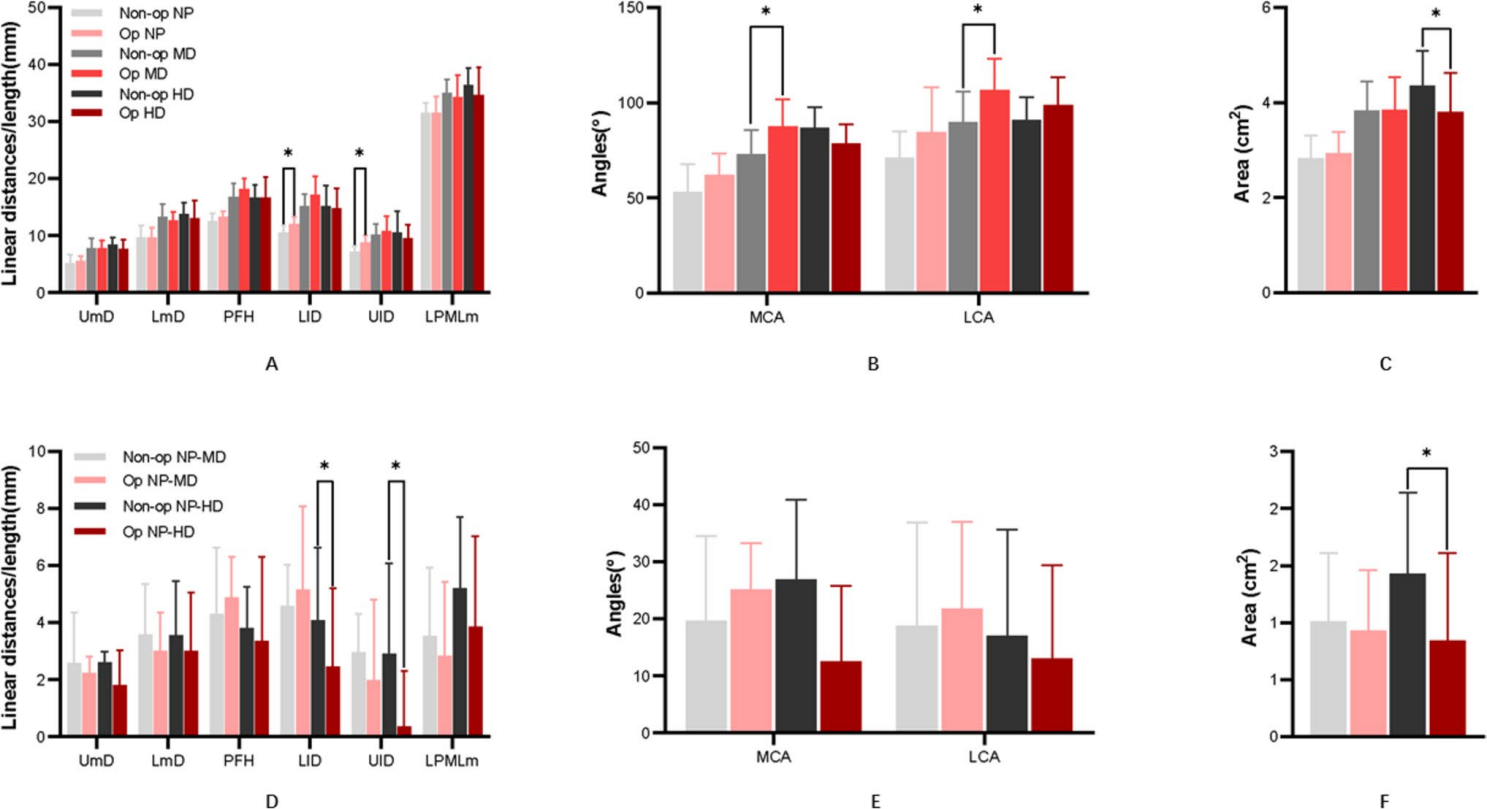
**A**–**F** Comparison of periocular measurements between the operated and contralateral non-operated eyes in patients with large lower eyelid defects (>50% of eyelid length) under different distraction conditions. **A**–**C** Absolute values of key measurements in neutral position (NP), manual distraction (MD), and hook distraction (HD). **D**–**F** Changes in measurements from neutral position to manual distraction (NP–MD) and hook distraction (NP–HD), illustrating differences in eyelid mobility and contour adaptation between eyes. *NP* neutral position, *MD* manual distraction, *HD* hook distraction. *p < 0.05; **p < 0.01; ***p < 0.001

Under NP, the lateral lid distance (LlD) and upper lateral lid distance (UlD) were significantly greater in the operated eyes (LlD: 12.00 ± 1.33 mm vs. 10.59 ± 1.27 mm, *p* = 0.018; UlD: 8.87 ± 1.14 mm vs. 7.23 ± 0.99 mm, *p* = 0.018). Similarly, under MD, the medial canthal angle (MCA) and lateral canthal angle (LCA) were significantly larger in the operated eyes (MCA: 87.54 ± 14.14° vs. 73.03 ± 12.93°, *p* = 0.018; LCA: 106.57 ± 16.68° vs. 90.03 ± 15.88°, *p* = 0.018) (Fig. 4A–C). However, the changes in measurements from NP to MD (MD–NP) did not show significant differences between operated and non-operated eyes (*p* > 0.1) (Fig. 4 D–F).

Under hook distraction (HD), most parameters were larger on the non-operated side compared to the operated side. Notably, the interpalpebral area was significantly greater on the non-operated side (4.36 ± 0.73 cm^2^ vs. 3.80 ± 0.83 cm^2^, *p* = 0.043) (Fig. 4A–C). Furthermore, when comparing changes from NP to HD (HD–NP), the non-operated side exhibited greater mobility, particularly in LlD, UlD, and interpalpebral area (LlD: 4.09 ± 2.55 mm vs. 2.47 ± 2.74 mm, *p* = 0.043; UlD: 2.92 ± 3.16 mm vs. 0.36 ± 1.95 mm, *p* = 0.043; area: 1.43 ± 0.71 cm^2^ vs. 0.84 ± 0.77 cm^2^) (Fig. 4D–F).

In patients with eyelid defects involving less than 50% of the lower eyelid length, periocular measurements demonstrated trends similar to those observed in the large defect group. Under neutral position (NP), most measurements were larger on the operated side compared to the non-operated side, with the lateral canthal angle (LCA) showing a statistically significant difference (82.59 ± 15.08° vs. 66.25 ± 14.70°, *p* = 0.038). This tendency persisted under manual distraction (MD), although without additional statistically significant differences.

**Fig. 5:**
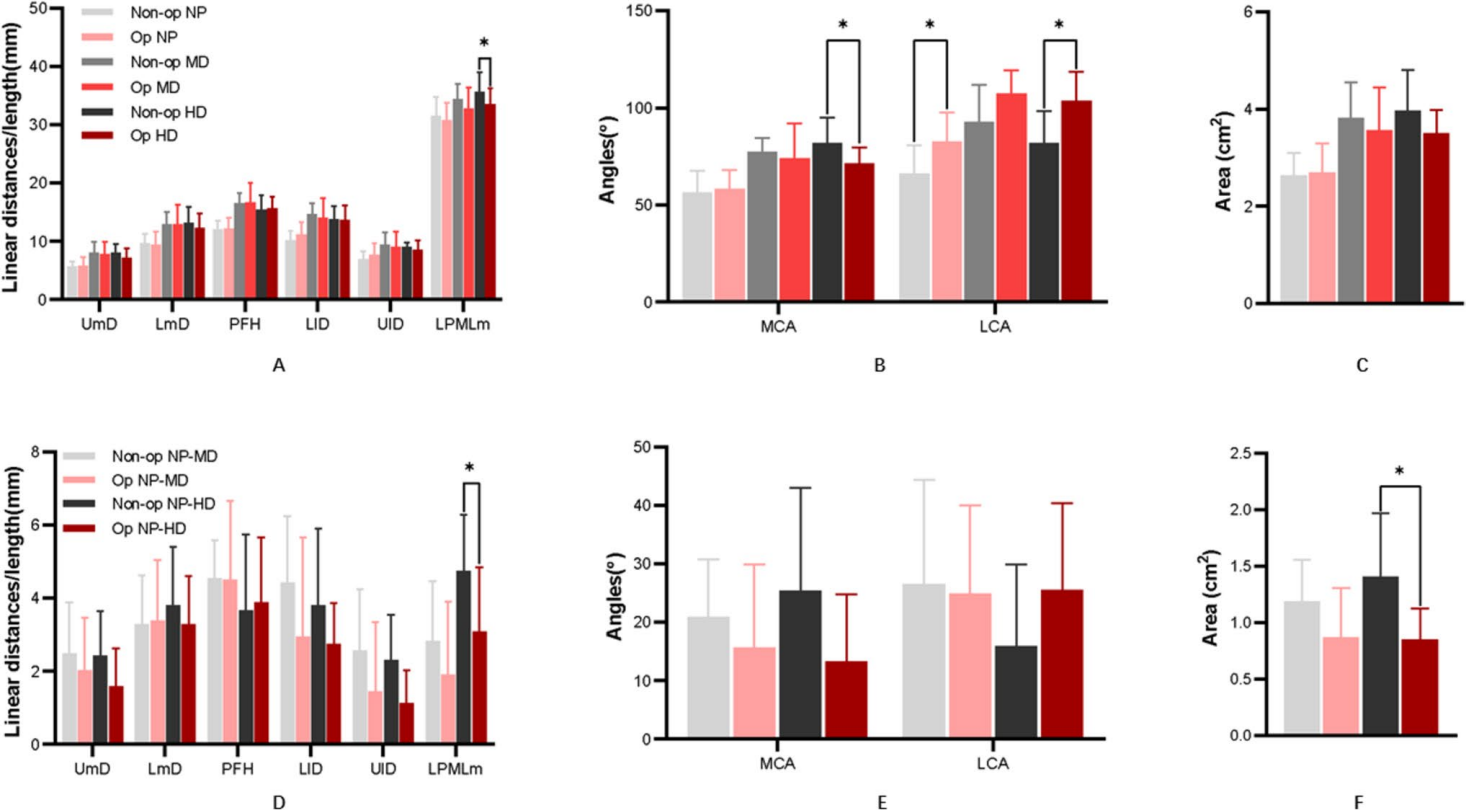
**A**–**F** Comparison of periocular measurements between the operated and contralateral non-operated eyes in patients with small lower eyelid defects (<50% of eyelid length) under different distraction conditions. **A**–**C** Absolute values of key parameters in neutral position (NP), manual distraction (MD), and hook distraction (HD). **D**–**F** Changes in measurements from neutral position to manual distraction (NP–MD) and hook distraction (NP–HD), demonstrating differences in eyelid mobility and contour between eyes. *NP* neutral position, *MD* manual distraction, *HD* hook distraction. *p < 0.05; **p < 0.01; ***p < 0.001

Under hook distraction (HD), most parameters were larger on the non-operated side, with significant differences observed in the lower palpebral margin length (LPMLm: 35.72 ± 3.36 mm vs. 33.52 ± 2.79 mm, *p* = 0.018) and the medial canthal angle (MCA: 82.07 ± 13.05° vs. 71.54 ± 8.23°, *p* = 0.028). An exception was noted in the LCA, which remained significantly larger on the operated side (103.63 ± 15.12° vs. 81.80 ± 16.48°, *p* = 0.018) (Fig. 5A–C).

When assessing changes from neutral position to hook distraction (HD–NP), the non-operated side exhibited more pronounced changes, particularly in LPMLm (4.76 ± 1.53 mm vs. 3.08 ± 1.77 mm, *p* = 0.028) and interpalpebral area (1.41 ± 0.56 cm^2^ vs. 0.85 ± 0.28 cm^2^, *p* = 0.018), mirroring the findings observed in patients with larger defects (Fig. 5D–F)

## Discussion

The Hughes tarsoconjunctival flap technique, through decades of clinical practice and refinement, has provided reliable functional and aesthetic outcomes with a low rate of secondary procedures, making it a widely accepted technique for full-thickness lower eyelid defect reconstruction [5, 12, 16, 17]. In facial and periocular morphology assessment, three-dimensional imaging has progressively supplanted traditional two-dimensional methods. Early studies predominantly employed landmark-based analysis under static conditions [14, 18], while contemporary evidence confirms the superior efficacy of stereophotogrammetry in evaluating lower eyelid laxity [14, 18, 19]. Systematic validation by Hou et al. established that the VECTRA M3 imaging system provides stable measurements across three states: natural position (NP), manual distraction (MD), and standardized hook distraction (HD), with the latter demonstrating optimal reproducibility due to its standardized protocol [13, 20].

This is the first study to apply three-dimensional anthropometry for dynamic assessment of post-Hughes procedure lower eyelid morphology. Leveraging the principle of bilateral periocular symmetry [21, 22], we innovatively utilized the contralateral non-operated eye as an internal control. Through systematic comparison of NP, standardized MD, and quantitative HD measurements, we achieved a comprehensive multiparametric evaluation of surgical outcomes.

Subgroup analysis by defect size provided further insights into how the extent of tissue excision influences postoperative morphology. As the Hughes flap is primarily indicated for defects exceeding 50% of eyelid length [23], our findings demonstrate larger defects result in more pronounced morphological changes, particularly in lateral eyelid distances and canthal angles, and reveal reduced eyelid mobility under distraction. These quantitative findings provide objective benchmarks for clinical assessment. Standardized weighted hook distraction demonstrated superior performance in postoperative evaluation, particularly for extensive defects. This aligns with Hou et al.’s conclusions [20], collectively supporting stereophotogrammetry’s utility in eyelid biomechanics research. However, the discrepancies noted between MD and HD, particularly in patients with larger defects, highlight the limitations of manual methods—likely attributable to differences in finger positioning, applied force direction, and patient-specific anatomical factors.

The Hughes procedure is technically complex and relatively uncommon, which restricted our study population to 16 patients who fulfilled strict inclusion criteria. Within this cohort, ectropion occurred in 57.1% of patients with large defects (>50%) but was absent in those with smaller defects. The occurrence of ectropion is likely associated with the extent of surgical reconstruction and resultant tissue tension; however, given the advanced mean age of our cohort, the potential contribution of age-related degenerative changes cannot be excluded. These findings highlight the need for more refined surgical strategies and tension-reducing techniques to minimize postoperative eyelid malposition, especially in extensive reconstructions. They also support developing modified approaches for elderly patients with large defects, potentially incorporating adjuncts such as fascial slings or biological mesh reinforcement. Despite prior evidence confirming the accuracy of the Vectra M3 system, some technical limitations must be acknowledged. Artifacts caused by lighting conditions, reflections, or surface irregularities can affect landmark precision and introduce measurement error [24]. Moreover, our study analyzed only postoperative images and relied on the assumption of bilateral periocular symmetry. However, natural facial asymmetry is common and may confound direct comparisons between eyes [25]. In addition, the relatively small sample size constitutes a major limitation of our study, as it reduces statistical power, restricts the generalizability of findings, and limits the ability to conduct more detailed subgroup analyses (e.g., by defect location or reconstruction method). This was primarily due to the low number of Hughes procedures performed during the study period. Furthermore, most patients were elderly, with some lost to follow-up due to unrelated causes such as death. The COVID-19 pandemic also contributed to reduced follow-up rates, as some patients and their families were reluctant to return for postoperative assessments. To address these limitations, future research could be improved in the following aspects: To enhance accuracy and minimize bias, future research should incorporate both pre- and postoperative imaging data and consider population-based asymmetry baselines when interpreting results, implement paired pre-operative baseline studies, develop region-specific algorithms for asymmetry correction, and conduct multicenter validation studies with larger sample sizes.

Although stereophotogrammetry enables objective and highly reproducible periocular measurements, many primary hospitals in under-resourced settings lack access to 3D scanners. Despite the demonstrated reliability of 3D facial biometric technology, it has not yet become a standard requirement in clinical practice. This technological gap presents a practical limitation. Without access to 3D instruments, users cannot benefit from their precision, efficiency, and intuitive visualization capabilities. However, the absence of 3D hardware does not render the underlying data or insights obsolete. Clinicians and researchers can still apply the principles derived from 3D studies using alternative approaches. For example, soft tissue deformation patterns in response to skeletal movements—characterized through 3D analysis—can inform and improve 2D-based predictive models. These insights can correct the inherent inaccuracies of 2D measurements and produce more reliable simulation outcomes. Additionally, telemedicine offers a viable alternative. Clinicians can transmit high-quality photographs (frontal, lateral, and oblique) and 2D radiographs to specialists equipped with 3D tools. These specialists can perform virtual 3D assessments and generate surgical recommendations based on their databases and experience. Primary centers can then apply these plans locally, effectively bridging the technology gap. By integrating 3D theoretical frameworks with real-world 2D resources and clinical expertise, practitioners in resource-limited settings can still extract value from 3D measurement research. This translational approach ensures that 3D-derived knowledge benefits broader clinical practice, even in the absence of advanced equipment.

The standardized 3D assessment framework serves dual purposes: not only does it provide an objective evaluation tool, but it also supplies critical data support for the predictive system. This comprehensive assessment system significantly contributes to surgical planning and postoperative evaluation while enabling effective long-term outcome monitoring. A postoperative outcome prediction system could be established upon prospective accumulation of larger-scale patient measurement data. Through precise pre-operative assessment of defect size and location, this system will facilitate personalized flap design to minimize postoperative complications and reduce adverse impacts on the ocular surface environment.

In conclusion, the Hughes procedure increases lower eyelid tension and reduces elasticity, particularly in the temporal region and in cases involving large defects. Three-dimensional anthropometric analysis under standardized distraction—especially with weighted hook techniques—provides a reliable method for objectively assessing postoperative eyelid morphology. These tools can inform surgical planning, postoperative evaluation, and long-term outcome monitoring. Future studies with larger, prospective cohorts and standardized pre-operative baselines are essential to validate these findings further and refine reconstructive strategies for lower eyelid defects.
